# NDRG4 promotes myogenesis via Akt/CREB activation

**DOI:** 10.18632/oncotarget.21591

**Published:** 2017-10-06

**Authors:** Mingfei Zhu, Rong Zheng, Yiwen Guo, Yunxia Zhang, Bo Zuo

**Affiliations:** ^1^ Key Laboratory of Swine Genetics and Breeding of Ministry of Agriculture & Key Laboratory of Agriculture Animal Genetics, Breeding and Reproduction of Ministry of Education, College of Animal Science and Technology, Huazhong Agricultural University, Wuhan 430070, P.R. China; ^2^ The Cooperative Innovation Center for Sustainable Pig Production, Wuhan 430070, China; ^3^ College of Life Science and Agronomy, Zhoukou Normal University, Zhoukou 466000, China

**Keywords:** myogenesis, NDRG4, CTMP, Akt, CREB

## Abstract

N-Myc downstream-regulated gene 4 (*NDRG4*) plays an important role in biological processes and pathogenesis, but its function in muscle development is unclear. In this study, we investigated the function of the *NDRG4* gene in the regulation of myogenic differentiation. NDRG4 expression is upregulated during muscle regeneration and C2C12 myoblast differentiation. Gain and loss of function studies revealed that NDRG4 dramatically promotes expression of myogenic differentiation factor (*MyoD*), myogenin (*MyoG*), and myosin heavy chain (*MyHC*) genes and myotube formation. Mechanistically, the binding of NDRG4 to carboxyl-terminal modulator protein (CTMP) abates the interaction of CTMP and protein kinase B (Akt) and increases the phosphorylation of Akt and cAMP response element binding protein (CREB), which leads to increased expression of myogenic genes. Our results reveal that NDRG4 promotes myogenic differentiation via Akt/CREB activation.

## INTRODUCTION

Muscle formation is a well-orchestrated process in which pluripotent mesodermal cells give rise to myoblasts, subsequently proliferate, withdraw from the cell cycle, and differentiate into myotubes [1]. Myogenesis is precisely regulated by myogenic regulatory factors (MRFs) such as myogenic factor 5 (*Myf5*), *MyoD*, *MyoG*, and myogenic factor 6 (*Myf6*; also known as *MRF4*) [2, 3]. *Myf5* and *MyoD* are primary MRF proteins expressed at the myoblast stage and are essential for skeletal muscle lineage determination [4]. *MyoD* overexpression alone is sufficient to reprogram fibroblasts into muscle cells [5, 6]. *MyoG* and *Myf6* are expressed upon differentiation of myoblasts into myotubes. Downregulation of *MyoG* can even reverse terminal muscle cell differentiation [7]. The activity of such factors is under tight posttranslational control by signal transduction pathways, including the Akt pathway [8]. Akt is activated during myogenic differentiation *in vitro* [9], and Akt overexpression enhances myoblast differentiation [10]. Akt knockout inhibits myofiber formation [11]. CREB is the downstream molecule of Akt and plays important roles in the differentiation of muscle cells [12–15]. The Akt/CREB signaling pathway is critical for muscle development and involves multiple proteins and molecules.

*NDRG4*, also known as *BDM1* and *SMAP-8*, is one of four members of the N-Myc downstream-regulated gene (NDRG) family, which is involved in cell proliferation, differentiation, development, and stress [16]. The NDRG family is highly conserved, and the identity between human and mouse NDRG4 is about 98%. Previous studies showed that *NDRG4* is highly expressed in the brain [17] and heart [18], and involved in brain and cardiac development [19, 20]. *NDRG4* is downregulated in numerous cancer cell lines and tumor types, and functions as a tumor suppressor gene [21–23]. A recent study demonstrated that estrogen stimulated *NDRG4* expression and that *NDRG4* expression levels are significantly upregulated at implantation sites during early pregnancy in mice. *NDRG4* plays critical roles in embryo implantation under the regulation of estrogen [24]. Previous gene expression omnibus (GEO) data (GDS586) show that *NDRG4* is expressed in non-differentiated myoblasts and is suddenly upregulated during C2C12 myogenic differentiation [25]. Furthermore, the GEO data (GDS4924) indicate that *NDRG4* expression levels are low in uninjured tibialis anterior (TA) muscle and strongly upregulated at 3 and 7 days after muscle injury by cardiotoxin (CTX) or glycerol. Interestingly, this expression pattern is almost identical to those of the *MyoD* and *MyoG* genes [26], suggesting that *NDRG4* may play a role in myogenic differentiation and skeletal muscle regeneration. Similar to NDRG4, MyoD, and MyoG activation, Akt and CREB are also activated during myogenic differentiation [8, 27] and skeletal muscle regeneration [15, 28]. Besides, the Search Tool for the Retrieval of Interacting Genes (STRING) is used to evaluate the relationship between *NDRG4* and Akt signaling pathway, and the result shows that NDRG4 is a gene in the Akt Signaling SuperPath. However, the effects of *NDRG4* on myoblast differentiation and the relationship between NDRG4 and the Akt/CREB signaling pathway are unclear.

In the present study, we investigated the function of the *NDRG4* gene in the regulation of C2C12 myogenic differentiation. Treatment of C2C12 myoblasts with NDRG4 activated the Akt/CREB signaling pathway and increased expression of the *MyoD* and *MyoG* genes, resulting in promotion of myoblast differentiation. Our findings define a novel function of the *NDRG4* gene in skeletal muscle development and suggest a molecular mechanism by which *NDRG4* promotes myogenic differentiation.

## RESULTS

### The *NDRG4* gene is upregulated during myogenic differentiation and skeletal muscle regeneration

**Figure 1:**
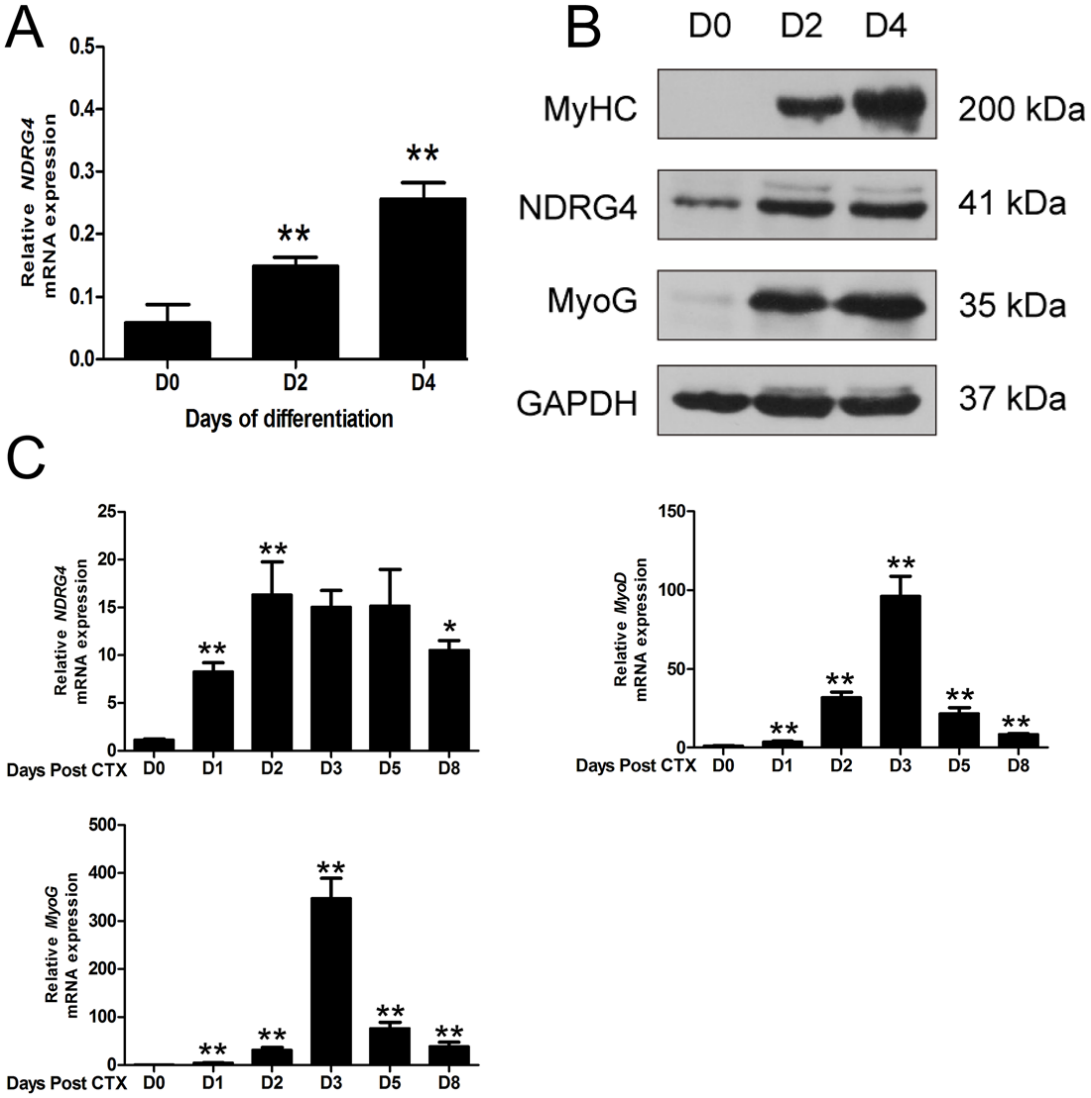
N-Myc downstream-regulated gene 4 (*NDRG4*) expression levels are increased during myoblast differentiation and muscle regeneration **(A)** qRT-PCR analysis of the expression of *NDRG4* in C2C12 cells at days 0, 2, and 4 after differentiation. **(B)** Western blot analysis of the NDRG4 protein in C2C12 cells at days 0, 2, and 4 after differentiation. Myogenin (MyoG) and MyHC are two marker genes of muscle differentiation. **(C)** qRT-PCR results showing the expression of the *NDRG4*, myogenic differentiation factor (*MyoD*), and *MyoG* genes during muscle regeneration. Hindlimb muscle was subjected to cardiotoxin (CTX) injection and harvested on the indicated days after injury for RNA analysis. GAPDH was used as an internal control. Values were normalized to GAPDH. Data are presented as means ± standard error of the mean (n = 3). Asterisks above columns represent significant differences among groups (^**^P < 0.01).

To investigate the role of NDRG4 in myoblast differentiation, qRT-PCR and Western blotting were used to determine the expression pattern of the *NDRG4* gene during C2C12 myoblast differentiation. The qRT-PCR results showed that expression of *NDRG4* increased gradually and significantly as C2C12 cells differentiated (Figure 1A). Western blotting results indicated that the NDRG4 protein was detected in proliferating myoblasts and that expression increased during myogenic differentiation, while the protein expression levels of two myogenic marker genes (MyoG and MyHC) increased greatly during myogenic differentiation of C2C12 cells (Figure 1B). As myogenic differentiation is an important event during skeletal muscle regeneration, we investigated the change in NDRG4 mRNA expression following CTX-mediated injury of mouse skeletal muscle. NDRG4 expression levels were low in uninjured hindlimb muscle and increased rapidly at 2 and 5 days after muscle injury by CTX. In this period, expression of both MyoD and MyoG also increased dramatically. Additionally, expression of NDRG4 peaked earlier than that of MyoD and MyoG (Figure 1C). Together, these results suggest that NDRG4 may be involved in myogenesis and muscle regeneration.

### The *NDRG4* gene had no effect on myoblast proliferation

**Figure 2:**
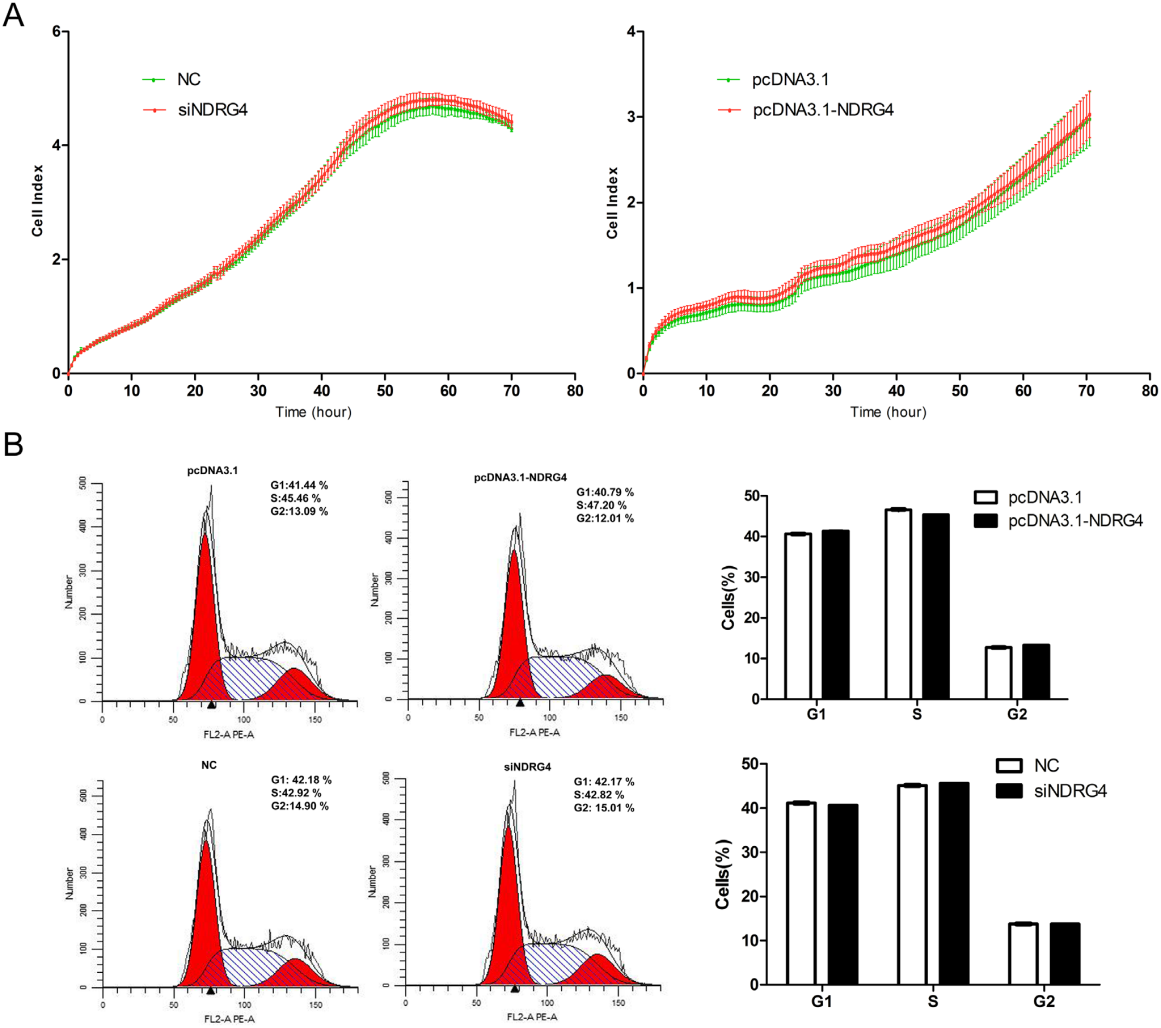
*NDRG4* has no effect on myoblast proliferation C2C12 cells were transfected with pcDNA3.1-NDRG4 or pcDNA3.1 and small interfering RNA against NDRG4 (siNDRG4) or negative control (NC). **(A)** Cell growth dynamics were continuously monitored by the xCELLigence system. **(B)** Cell cycle phase was analyzed 48 h after transfection by propidium iodide flow cytometry.

As the *NDRG4* gene regulates the proliferation of cancer-associated cells, we performed gain- and loss-of-function studies to examine whether NDRG4 has the same roles in myoblast proliferation. C2C12 cells were transfected with the pcDNA3.1-NDRG4 or pcDNA3.1 plasmid and pooled siNDRG4 or negative control (NC). When the cell index reached about 1.0, cell growth dynamics were continuously monitored by an xCELLigence system and the cell cycle was examined by flow cytometry. No significant differences in cell growth rates or the cell cycle were observed between the treated cells and the control (Figure 2A and 2B). These observations indicated that NDRG4 had no effect on the proliferation of C2C12 cells.

### Knockdown of *NDRG4* inhibits myogenic differentiation

**Figure 3:**
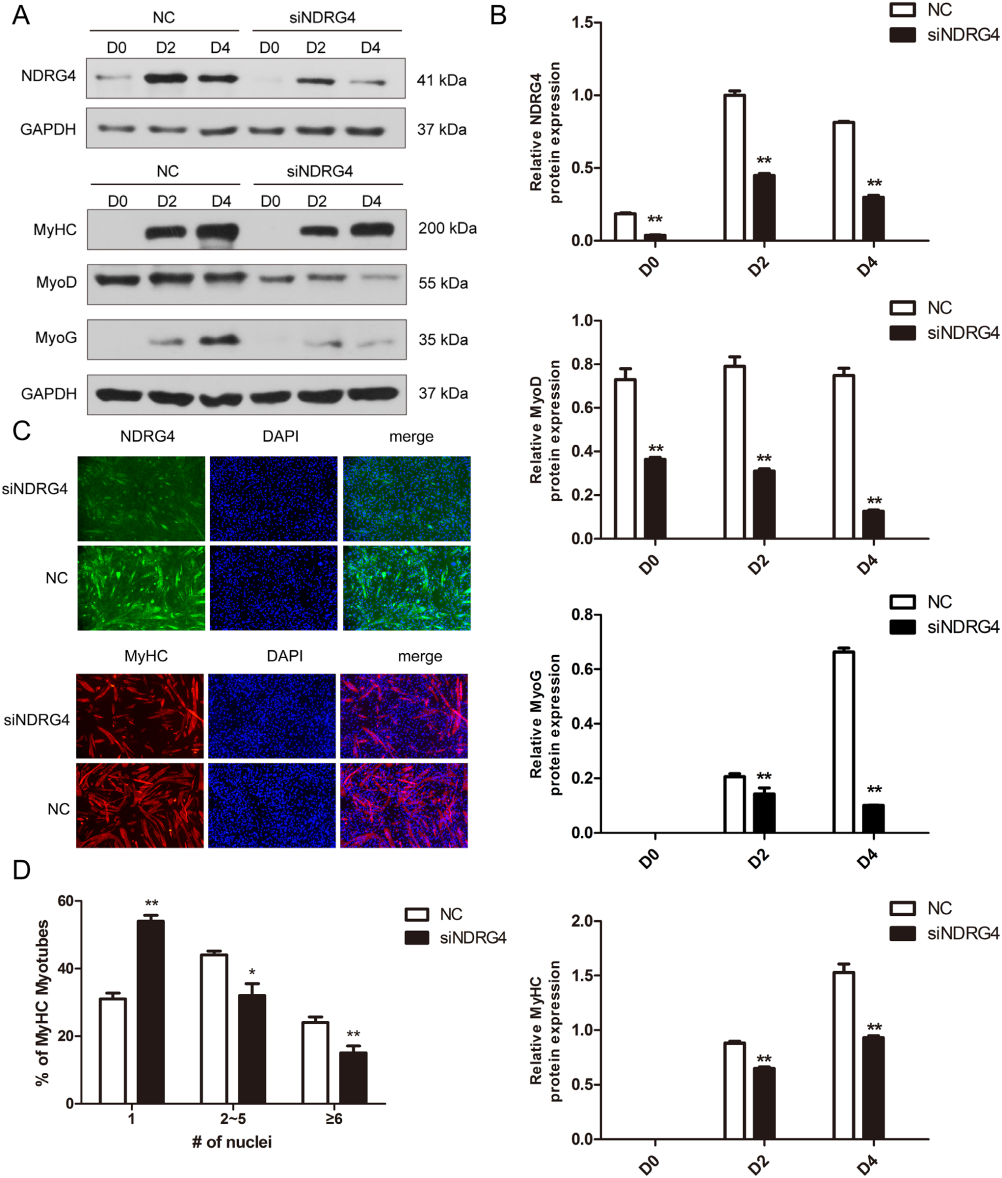
Knockdown of *NDRG4* inhibits myogenic differentiation **(A)** Western blot results of NDRG4, MyoD, MyoG, and MyHC protein expression levels after NDRG4 knockdown at 0 days (D0), 2 days (D2), and 4 days (D4). **(B)** Relative protein expression levels represented by the ratio of detected protein to the GAPDH protein expression level after NDRG4 knockdown at D0, D2, and D4. The quantifications of Western blot data are presented as means ± standard deviation (SD; n = 3). **(C)** Immunofluorescence results of NDRG4 and MyHC protein expression levels after NDRG4 knockdown. Transfected C2C12 myoblasts were differentiated for 5 days, and stained with anti-NDRG4 (green) and anti-MyHC antibodies (red), and 4’,6-diamidino-2-phenylindole (DAPI; blue), and imaged by fluorescence microscopy. Bars, 200 μm. **(D)** The quantification of myotube formation is shown in the panel.

Since NDRG4 expression was increased during myogenesis and the expression of the NDRG4 protein was positively correlated with the levels of myogenic markers (MyoG, MyHC) during myogenic differentiation, as mentioned above, we speculated that NDRG4 may have a positive role in C2C12 cell differentiation. To test this hypothesis, loss-of-function studies were performed. The cells were lysed at 0, 2, and 4 days after cell differentiation. The results demonstrated that NDRG4 siRNA significantly inhibited the protein expression of NDRG4 at day 0 (D0), day 2 (D2), and day 4 (D4) post transfection. Meanwhile, the expression levels of MyoD, MyoG, and MyHC were significantly suppressed (Figure 3A and 3B). The immunofluorescence of MyHC was used to examine myotube formation in C2C12 cells 5 days after transfection. MyHC-positive cells were scored as mononucleate, containing two to five nuclei, or containing six or more nuclei. NDRG4 knockdown cells formed smaller myotubes with more mononucleate cells (54%) compared to control cells (31%). In contrast, the percentage of myotubes containing cells with two to five nuclei and six or more nuclei decreased to 32% and 14%, compared to 45% and 24% of control cells, respectively (Figure 3C and 3D). These results clearly demonstrate that NDRG4 knockdown inhibited myoblast differentiation *in vitro*.

### Overexpression of *NDRG4* enhances myogenic differentiation

**Figure 4:**
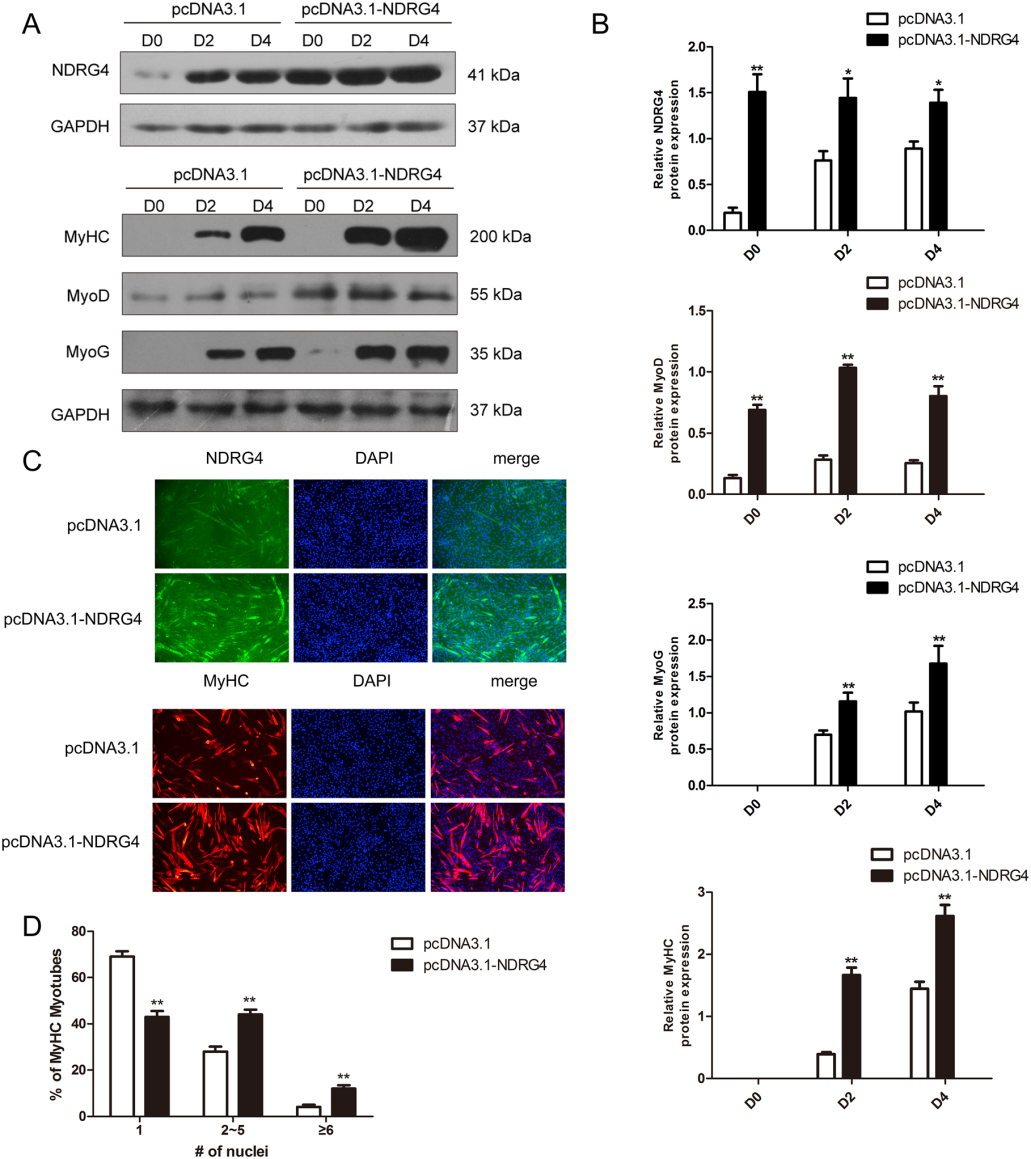
Overexpression of *NDRG4* enhances myogenic differentiation **(A)** Western blot results of NDRG4, MyoD, MyoG, and MyHC protein expression levels after NDRG4 overexpression at D0, D2, and D4. **(B)** Relative protein expression levels represented by the ratio of detected protein to GAPDH protein expression level after NDRG4 overexpression at D0, D2, and D4. **(C)** Immunofluorescence results of NDRG4 and MyHC protein expression levels after NDRG4 overexpression. Transfected C2C12 myoblasts were differentiated for 3 days, stained with anti-NDRG4 (green) and anti-MyHC antibodies (red), and DAPI (blue), and imaged by fluorescence microscopy. Bars, 200 μm. **(D)** The quantification of myotube formation is shown in the panel.

To confirm the above results, C2C12 myoblasts were transfected with control or NDRG4 expression vectors and induced to differentiate. Total protein was extracted at D0, D2, and D4. The expression level of NDRG4 was significantly increased with overexpression, and the expression of myogenic marker genes, including MyoD, MyoG, and MyHC, was significantly enhanced in *NDRG4*-overexpressing C2C12 myoblasts compared to the control (Figure 4A and 4B). Next, we examined the effect of NDRG4 overexpression on myotube formation. Control and NDRG4-overexpressing C2C12 cells were induced to differentiate for 3 days and stained with anti-MyHC antibody followed by 4’,6-diamidino-2-phenylindole (DAPI) staining. NDRG4-overexpressing C2C12 cells formed larger and more numerous myotubes than control (pcDNA3.1) cells (Figure 4C and 4D). These data suggest that NDRG4 promotes myoblast differentiation.

### NDRG4 activates the Akt/CREB pathway in myoblast differentiation

**Figure 5:**
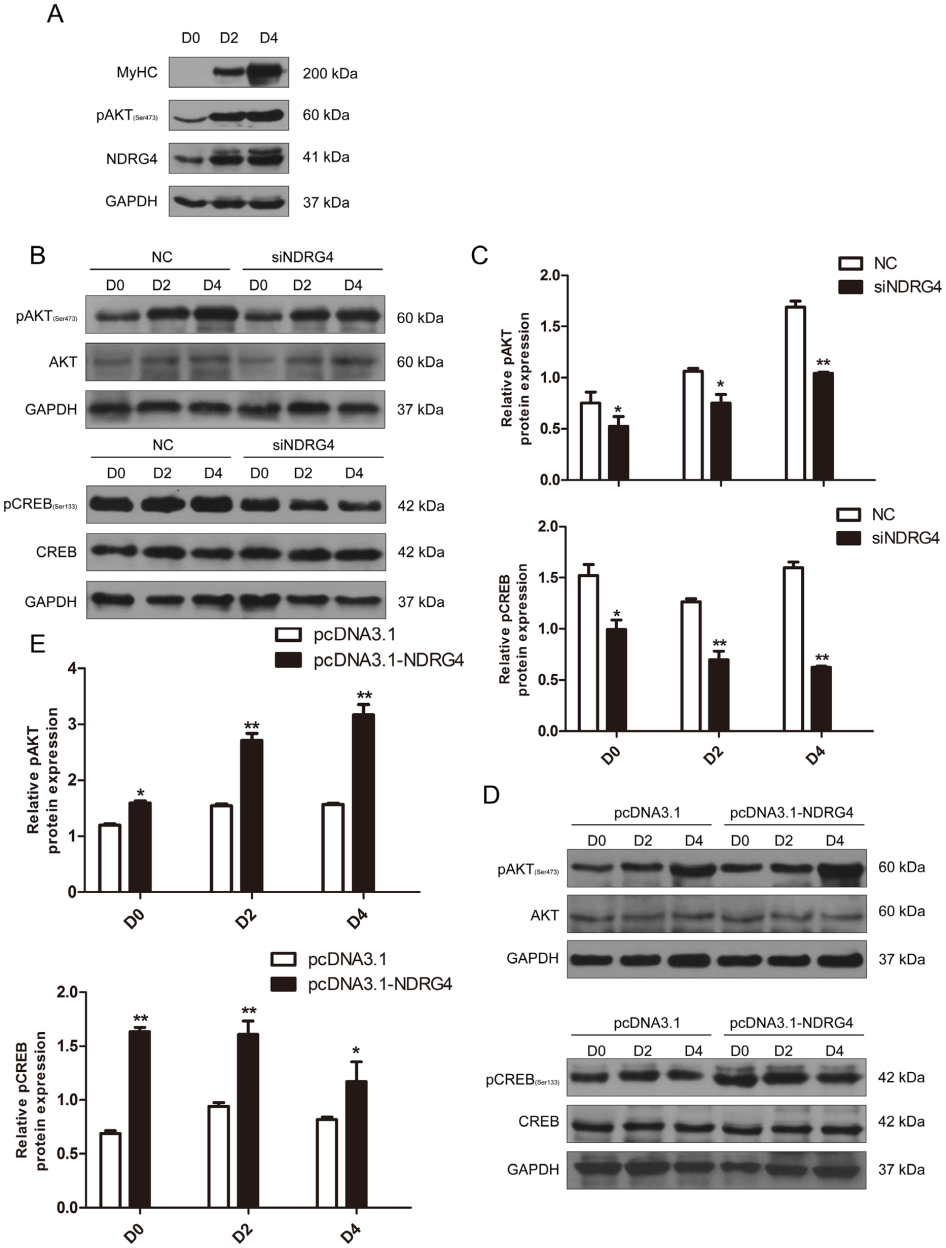
NDRG4 activates the Akt/CREB pathway during myoblast differentiation **(A)** Western blot results of NDRG4 and pAkt protein expression levels in C2C12 cells at differentiation days 0, 2, and 4. MyoG and MyHC are two known markers of muscle differentiation. **(B)** Western blot results of pAkt, Akt, pCREB, and CREB protein expression levels in C2C12 cells transfected with siNDRG4. **(C)** Relative protein expression levels of pAkt and pCREB after NDRG4 knockdown in C2C12 cells at D0, D2, and D4. **(D)** Western blot results showing the changes in pAkt, Akt, pCREB, and CREB protein expression levels in C2C12 cells transfected with NDRG4 plasmids. **(E)** Relative protein levels of pAkt and pCREB after NDRG4 overexpression at D0, D2, and D4. ^*^P < 0.05; ^**^P < 0.01.

To determine the molecular pathways by which NDRG4 mediates myogenic differentiation, we selected Akt as a candidate for further analysis, as it is a well-known mediator of cell survival [29, 30] and a key regulator of myogenic differentiation [8] and muscle mass [31]. First, we analyzed Akt phosphorylation in myogenesis, and found that the phosphorylation level gradually increased during myoblast differentiation, which was similar to the expression pattern of NDRG4 (Figure 5A). Next, we examined whether the Akt signaling pathway is involved in NDRG4 regulated myogenesis. As expected, significant decreases in phosphorylated Akt expression were observed in NDRG4 knockdown C2C12 cells compared with the control (Figure 5B and 5C, upper), while NDRG4 overexpression had the opposite effect (Figure 5D and 5E, upper). Taken together, these results suggest that NDRG4 participates in Akt-mediated myogenic differentiation. Second, CREB was chosen for further study, as it is a key regulatory target of Akt and closely associated with myogenesis. Overexpression of NDRG4 enhanced CREB activation as determined by an increase in CREB phosphorylation at Ser133, but did not significantly alter the expression of CREB (Figure 5D and 5E, lower). NDRG4 knockdown had the inverse effect (Figure 5B and 5C, lower), confirming the results of the overexpression experiment. These results suggest that NDRG4 promotes C2C12 myogenic differentiation by activating the Akt/CREB signaling pathway.

### NDRG4 and CTMP physically interact in differentiating myoblasts and the interaction mediates the activity of Akt

**Figure 6:**
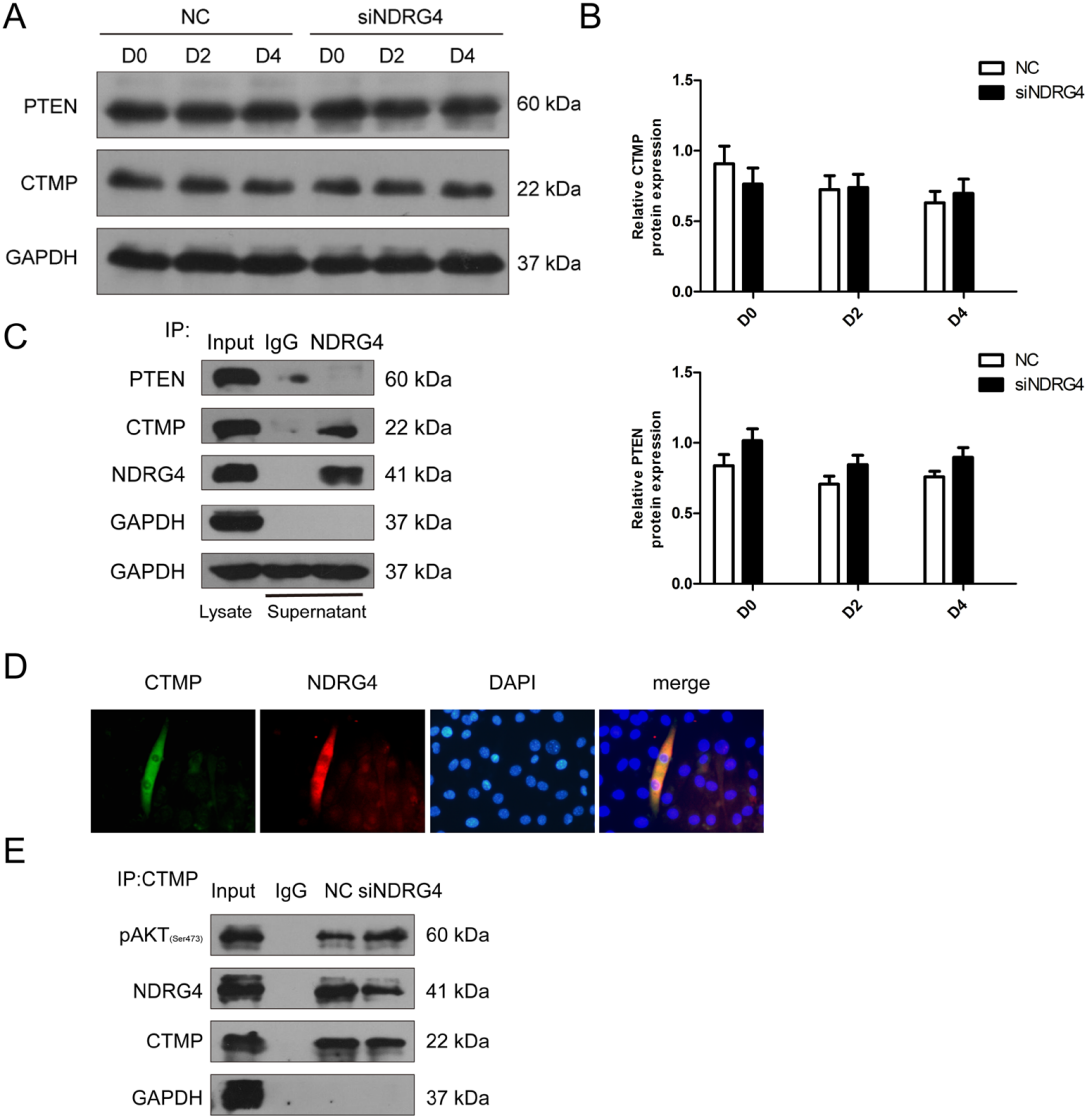
NDRG4 and CTMP physically interact in differentiating myoblasts and this interaction mediates Akt activity **(A)** Western blot results showing the changes in phosphatase and tensin homolog (PTEN) and CTMP protein expression levels after NDRG4 knockdown. **(B)** Relative protein expression levels of CTMP and PTEN represented by the ratio of detected protein to GAPDH protein expression level after NDRG4 knockdown at D0, D2, and D4. **(C)** Co-immunoprecipitation results of the interaction between NDRG4 and CTMP *in vivo*. C2C12 cells were differentiated for 3 days, harvested, and pulled-down by antibodies. Lane 1: The cell lysate (Input) was used as a positive control for Western blotting analysis. Lane 2: Immunoprecipitation results with anti-IgG antibody as a negative control. Lane 3: Immunoprecipitation results with anti-NDRG4 antibody. **(D)** The subcellular localization of NDRG4 and CTMP demonstrated by immunofluorescence in C2C12 myotubes. C2C12 myotubes were stained with antibodies against NDRG4 (red), CTMP (green), and DAPI (blue). **(E)** NDRG4-knockdown C2C12 cells and control cells were lysed and immunoprecipitated with either CTMP antibody or control IgG. Immuno-complexes and input cell lysates were analyzed by Western blotting with anti-pAkt, anti-NDRG4, and anti-CTMP antibodies.

To investigate further the mechanism by which NDRG4 enhances the phosphorylation levels of Akt, we attempted to identify the Akt-interacting proteins phosphatase and tensin homolog (PTEN) and CTMP. Akt phosphorylation is negatively regulated by PTEN [32], which contributes to cell growth and proliferation. CTMP is an Akt-binding protein and suppresses Akt activity by inhibiting Akt phosphorylation [33]. Western blotting was performed to determine expression of PTEN and CTMP after *NDRG4* knockdown, but no significant differences in expression were found (Figure 6A and 6B). Previous studies have shown that NDRG4 can recruit some proteins to play biological functions [34, 35]. Thus, we examined whether there was any physical interaction between NDRG4 and PTEN or CTMP in C2C12 cells. C2C12 cells were induced to differentiate and the lysates were immunoprecipitated with control IgG or NDRG4 antibodies followed by immunoblotting with PTEN and CTMP antibodies, only CTMP strongly co-immunoprecipitated with NDRG4 (Figure 6C). Immunofluorescence confocal microscopic analysis was employed to determine the subcellular location of NDRG4 and CTMP in C2C12 cells. The results revealed that NDRG4 co-localized with CTMP in the cytoplasm (Figure 6D). These findings suggest that NDRG4 interacts with CTMP under basal conditions. It was reported that binding of CTMP to pAkt reduces Akt activity by inhibiting phosphorylation of serine 473 and threonine 308. We next investigated whether NDRG4 affected the binding of CTMP to pAkt during myogenic differentiation. We found that knockdown of NDRG4 decreased the interaction between NDRG4 and CTMP during C2C12 differentiation compared to that of control cells, while the binding capacity of CTMP to pAkt increased (Figure 6E). Simultaneously, NDRG4 knockdown decreased expression of phosphorylated Akt in C2C12 cells (Figure 5B). Collectively, these observations indicate that NDRG4 and CTMP physically interact in differentiating myoblasts, and this interaction increases the phosphorylation of Akt.

### NDRG4 promotes myogenesis by improving CREB transcription activity

**Figure 7:**
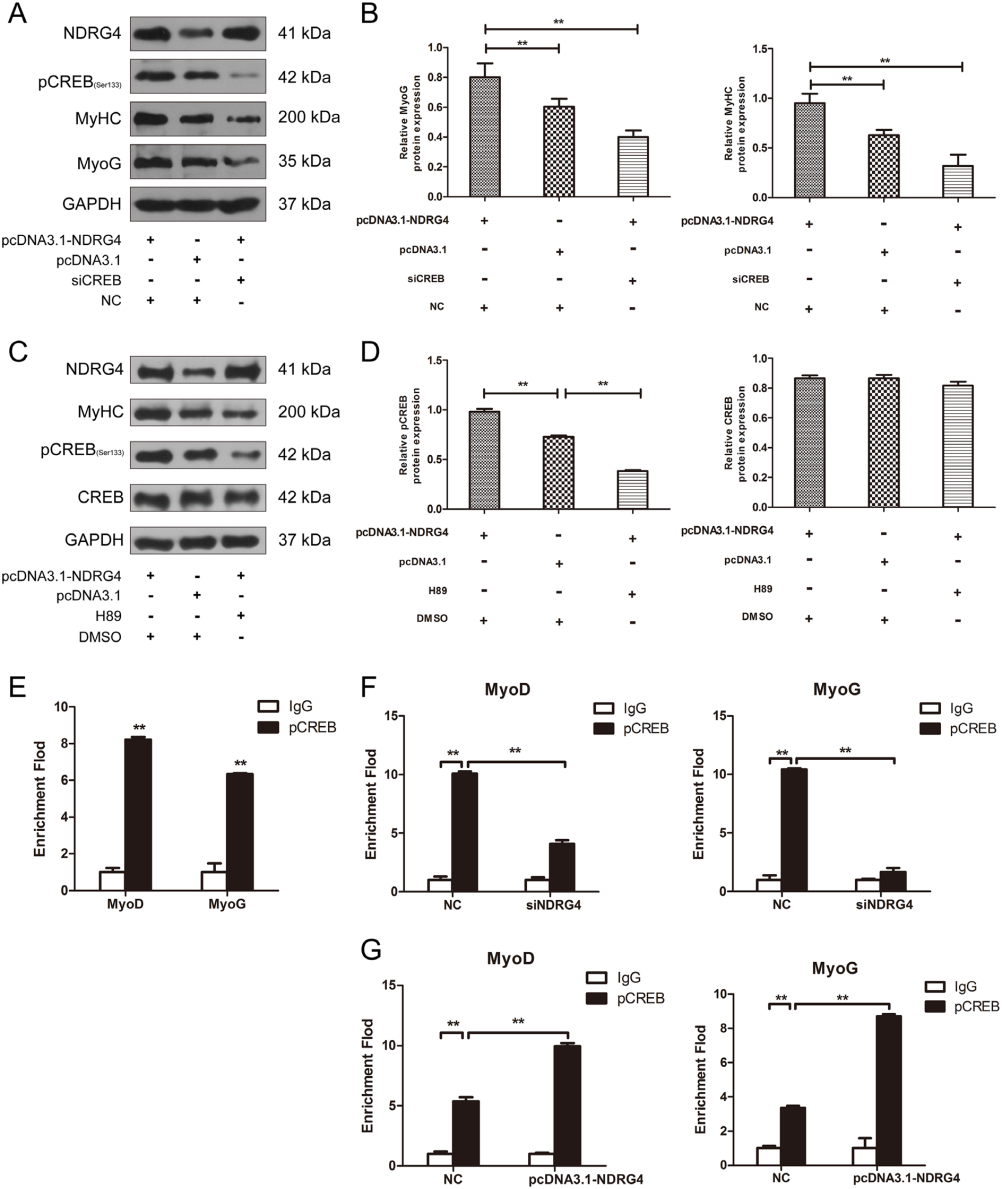
NDRG4 promotes myogenesis by increasing CREB transcription activity **(A)** Western blot results of MyoG and MyHC protein expression levels in C2C12 myoblasts after co-transfection with pcDNA3.1-NDRG4 and siCREB and differentiation for 3 days. **(B)** Relative protein expression levels of MyoG and MyHC after co-transfection with pcDNA3.1-NDRG4 and siCREB. **(C)** NDRG4 promotes myogenesis through pCREB. After NDRG4 overexpression, C2C12 cells were treated or not with 10 μM H89 and differentiated for 3 days, Protein expression levels of MyHC, CREB and pCREB were detected by western blotting. **(D)** Relative protein expression levels of CREB and pCREB after NDRG4 overexpression and H89 treatment. **(E)** Chromatin immunoprecipitation (ChIP) assay results showing the binding of pCREB to the MyoD and myogenin promoters *in vivo* in C2C12 myoblasts differentiated for 3 days. Normal mouse IgG was used as the negative control (as below). **(F)** ChIP assay results showing the effects of NDRG4 knockdown on the binding capacity of pCREB to the MyoD and MyoG promoters in siNDRG4 and NC cells cultured in differentiation medium for 3 days. **(G)** ChIP assay results showing the effects of NDRG4 overexpression on the binding capacity of pCREB to the MyoD and MyoG promoters in pcDNA3.1 and pcDNA3.1-NDRG4 cells cultured in differentiation medium for 3 days.

To confirm that NDRG4 promotes myogenesis through CREB, pcDNA3.1-NDRG4 and siCREB were co-transfected in C2C12 myoblasts. Overexpression of NDRG4 promoted MyoG and MyHC protein expression. After transfection with CREB siRNA fragments, overexpression of NDRG4 did not increase the protein expression of MyoG and MyHC compared with the control (Figure 7A and 7B). To detect whether NDRG4 was involved in CREB phosphorylation-mediated myogenesis, we used the phosphorylation inhibitor H89 to decrease the CREB phosphorylation level [36, 37], after overexpression of NDRG4. The results are similar to those of the RNA interference experiments, nevertheless, it did not significantly alter the expression of CREB (Figure 7C and 7D). Therefore, the effect of NDRG4 on myogenesis was dependent on CREB protein phosphorylation. Previous studies showed that CREB is a transcription factor that binds to cAMP response elements (CRE) in the regulatory region of target genes [36, 38, 39]. To investigate whether CREB directly regulates myogenic genes by binding to their promoter regions, we used the gene-regulation database (http://gene-regulation.com/pub/programs/alibaba2.html) to predict putative CRE-like binding sites. A ChIP assay was performed based on the predicted sites. The results showed that CREB can directly bind the promoter region of *MyoD* and *MyoG* genes (Figure 7E). Knockdown of *NDRG4* decreased the binding capacity of CREB to the CRE of *MyoD* and *MyoG* promoters (Figure 7F). Meanwhile, NDRG4 overexpression had the inverse effect (Figure 7G). These results suggest that NDRG4 strengthens C2C12 myogenic differentiation by improving CREB transcription activity at the promoters of its target genes.

## DISCUSSION

The NDRG4 protein plays important roles both *in vitro* and *in vivo*. Knockdown of the *NDRG4* gene dramatically decreases cell viability of glioblastoma cells [21]. The proliferation rate of A10 cells is significantly decreased after overexpression of *NDRG4* [40]. Schwann cell proliferation does not significantly differ between *NDRG4* morphant embryos and controls throughout development [41]. Here, we first confirmed that *NDRG4* is a positive regulator of myogenic differentiation, but *NDRG4* has no effects on myoblast proliferation and the cell cycle. These results showed that the effects of the *NDRG4* gene on cell phenotype depend on cellular context. *In vivo*, NDRG4 knockdown in zebrafish embryos causes a marked reduction in proliferative myocytes and results in hypoplastic hearts [20]. Targeted disruption of the NDRG4 gene in mice results in abnormalities in the spleen and thymus, as well as neurological defects [42]. NDRG4 protein-deficient mice exhibit spatial learning deficits and vulnerabilities to cerebral ischemia [19]. Previous studies suggested that NDRG4 was predominantly expressed in the brain and heart of postnatal rats [43]. The function of *NDRG4* in skeletal muscle is less well known. We confirmed that expression of *NDRG4* increases gradually in the early stages of muscle regeneration. Additionally, expression of pig *NDRG4* is significantly higher in embryonic muscle (35, 63, and 91 dpc) than in adult muscle [44]. Therefore, it can be speculated that *NDRG4* plays an important role in skeletal muscle determination and early development.

Akt/CREB is a classic signaling pathway involved in numerous biological processes. Nobiletin protects the brain from ischemic damage by activating the Akt/CREB signaling pathway [45]. Combined treatments with Ampakine and brain-derived neurotrophic factor (BDNF) enhance post stroke functional recovery in aged mice via Akt-CREB signaling [46]. Nexrutine® inhibits prostate cancer cell proliferation through modulation of Akt and CREB-mediated signaling pathways [47]. During muscle development, Akt is a key regulator of myogenesis [8] and is essential for muscle regeneration [28]. CREB is activated after muscle injury and can promote myogenesis and muscle regeneration [15]. Here, we confirmed that NDRG4 induced Akt and CREB phosphorylation, enhanced the binding capacity of pCREB to CREs in the *MyoD* and *MyoG* genes, and promoted myogenic differentiation and myotube formation. Previous studies showed that Akt regulates skeletal muscle hypertrophy *in vivo* via two signaling pathways [48–50]. One is the mechanistic target of rapamycin (mTOR) pathway, which controls protein synthesis [51, 52], and the other is the forkhead box O (FoxO) pathway, which controls protein degradation [53]. It would be interesting to determine whether NDRG4 affects these signaling pathways.

A previous report showed that interaction between blood vessel epicardial substance (Bves) and NDRG4 influenced epicardial cell movement [35]. NDRG4 directly interacts with p53 in malignant meningioma cells [34]. In this study, we found that NDRG4 interacts with CTMP during C2C12 cell differentiation. CTMP directly binds pAkt and decreases the phosphorylation of Akt [33, 54]. We confirmed that binding of NDRG4 to CTMP weakened the association of CTMP and pAkt, and thereby altered the phosphorylation of Akt. Based on our results, we speculate that increased NDRG4 during myogenesis antagonizes the inhibitory effect of CTMP on Akt phosphorylation. CTMP protein is comprised of a catalytically active hotdog-fold acyl-CoA thioesterase domain and an N-terminal domain, and the N-terminal domain is partially composed of an irregular and flexible secondary structure, reminiscent of a protein-binding domain [55]. Therefore, we assume that NDRG4 competes with Akt to bind to the N-terminal domain of CTMP. The NDRG4 protein has NDR- and α/β hydrolase-fold motifs. Additionally, it also contains one phosphorylation site and seventeen acetylation sites [16]. Beyond that, little is known about the relationship between NDRG4 protein structure and its biological function. Therefore, further investigations are needed to clarify which domain of NDRG4 is necessary and sufficient for its function as a positive regulator during myogenic differentiation.

**Figure 8:**
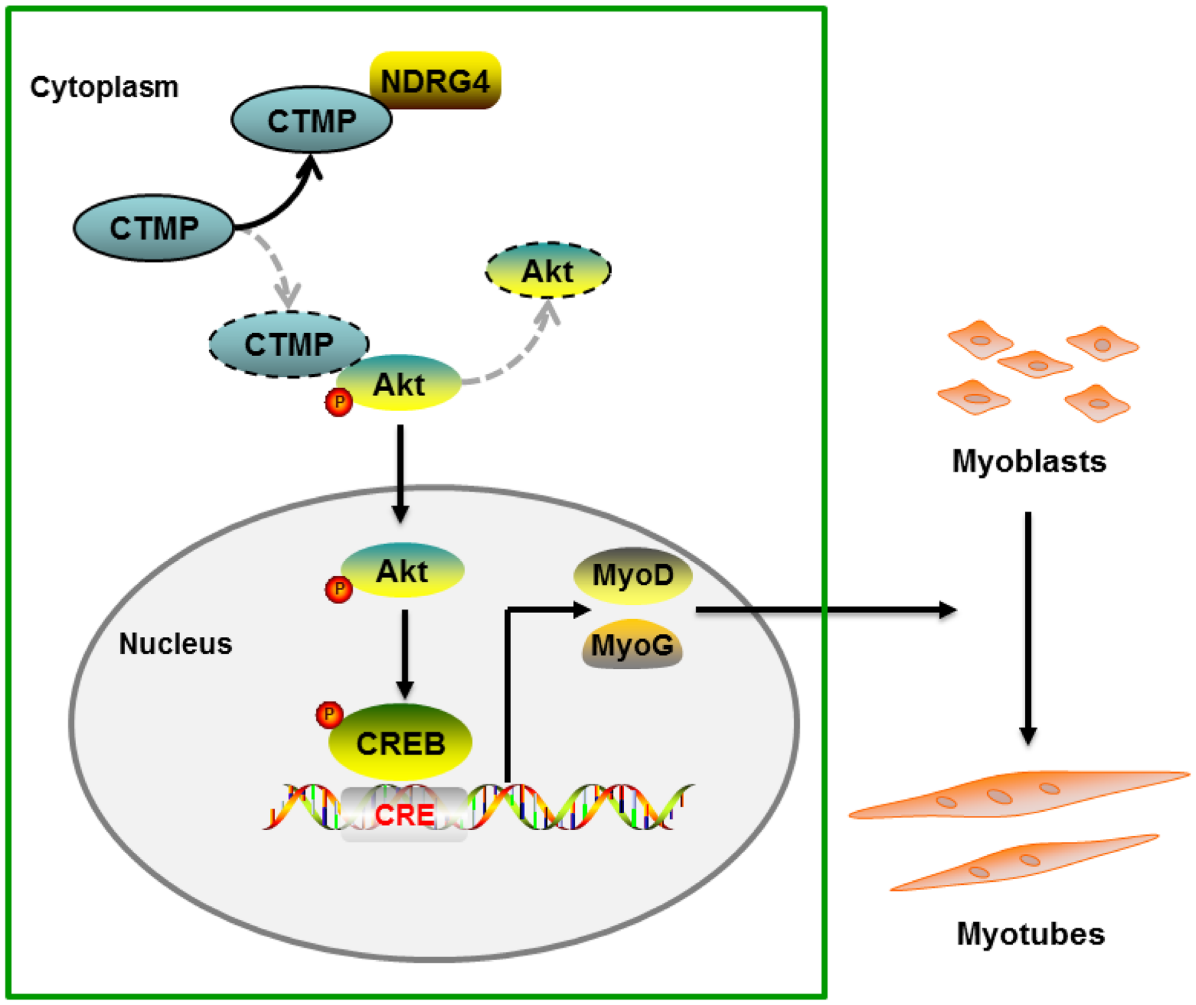
Proposed pathway model for NDRG4 involved in myoblast differentiation Binding of NDRG4 to CTMP attenuates the inhibitory effect of CTMP on Akt phosphorylation and further promotes the phosphorylation of CREB, leading to increased expression of the *MyoD* and *MyoG* genes and promotion of myoblast differentiation.

In summary, we showed that NDRG4 promotes myogenic differentiation through the Akt/CREB signaling pathway. The mechanism of action is the binding of NDRG4 to CTMP, which abates the interaction of CTMP and pAkt, increasing the phosphorylation level of Akt (Figure 8). Our results suggest that NDRG4 plays an important role in regulation of myogenesis during skeletal muscle development.

## MATERIALS AND METHODS

### Murine model of CTX-induced muscle injury

Muscle injury was performed by injecting 100 μL of 10 μM CTX (Sigma-Aldrich, St. Louis, MO, USA) into the hindlimb muscles of 8-week-old C57BL/6 mice. Muscles were harvested at days 0, 1, 2, 3, 5, and 8 after injection for RNA extraction.

### Cell culture and differentiation of C2C12 myoblasts

Mouse C2C12 cells were cultured in growth medium (GM) (Dulbecco's modified Eagle medium [DMEM]; Hyclone, Marlborough, MA, USA) supplemented with 10% fetal bovine serum (Gibco, Australia) and were maintained at 37°C in a 5% CO_2_ incubator. For differentiation experiments, when cells reached 80–90% confluence, the medium was changed to diffrentiation medium (DM) (DMEM supplemented with 2% horse serum [Gibco]). Differentiation medium was replaced every 24 h.

### Plasmids, small interfering RNAs (siRNAs), and transfection

For NDRG4 expression vector, mouse NDRG4 CDS was amplified by NDRG4-F (5’-CCCAAGCTTATGCCGGAGTGCTGGGATGG-3’) and NDRG4-R (5’-GGAATTCTCAGCAGGACACCTCCATGG-3’) primers and inserted into pcDNA3.1 vector. NDRG4 siRNAs (S: GAUGCUGGUAGUCGGAGAUAATT; A: UUAUCUCCGACUACCAGCAUCTT) and CREB siRNAs (S: GGACCUUUA CUGCCACAAATT; A: UUUGUGGCAGUAAAGGUCCTT) were synthesized by GenePharma (Shanghai, China). C2C12 cells were transfected with plasmids or siRNAs using Lipofectamine 2000 transfection reagent (Invitrogen, Carlsbad, CA, USA) according to the manufacturer's instructions. Opti-MEM I Reduced Serum Medium (Gibco) was used to dilute Lipofectamine 2000 and nucleic acids. All transfections were performed in triplicate for each experiment.

### Cell proliferation analysis

C2C12 cells were seeded on a 16-well E-Plate at 5000 cells per well and allowed to grow for 12–24 h. The cells were transfected with NDRG4 expression vector or siNDRG4 when the cell index reached about 1.0 in at least three wells per treatment. Cell growth and proliferation were monitored by an xCELLigence RTCA DP instrument (Roche Applied Science, Penzberg, Upper Bavaria, Germany).

The cell cycle was analyzed by flow cytometry. Briefly, 48 h after transfection, the C2C12 cells were fixed in 70% (v/v) ethanol overnight at −20°C. Following incubation in 50 mg/ml propidium iodide (PI) for 30 min at 4°C, cell cycle profiling was performed using a FACSCalibur Flow Cytometer (Becton Dickinson, Franklin Lakes, NJ, USA) and the data were analyzed using ModFit software (Verity Software House).

### RNA isolation and quantitative real-time PCR (qRT-PCR)

Total RNA from C2C12 myoblasts and skeletal muscle was extracted using an HP Total RNA Kit (Omega Bio-tek, Norcross, GA, USA) and treated with DNase I (Thermo Fisher Scientific, Waltham, MA, USA). The concentration and quality of RNA were assessed using a NanoDrop 2000 (Thermo Fisher Scientific) and agarose gel electrophoresis. Total RNA was reverse transcribed using the PrimeScript RT reagent kit with genomic DNA eraser (Takara, Japan). The qRT-PCR reaction was performed in a LightCycler 480 II (Roche, Basel, Switzerland) system using the SYBR® Green Real-time PCR Master Mix (Toyobo, Japan). The Ct (2^–ΔΔCt^) method was used to analyze the relative gene expression data following the literature.

### Western blotting

Cells were lysed in RIPA lysis buffer (Beyotime, Jiangsu, China) according to the manufacturer's instructions. After being mixed with sodium dodecyl sulfate (SDS) sample buffer and boiled for 10 min, denatured protein samples were separated by 10% SDS-polyacrylamide gel electrophoresis and electrotransferred onto polyvinylidene fluoride membranes (Millipore, Billerica, MA, USA) using a Mini Trans-Blot Cell system (Bio-Rad, Hercules, CA, USA). The membranes were blocked with 5% non-fat milk for 2 h and incubated overnight at 4°C with primary antibodies against NDRG4 (Santa Cruz Biotechnology, Santa Cruz, CA, USA; sc-166,917, 1:200 dilution), MyoG (Santa Cruz Biotechnology; sc-12,732, 1:200 dilution), MyHC (Santa Cruz Biotechnology; sc-376,157, 1:3000 dilution), CREB (Cell Signaling Technology, Danvers, MA, USA; 48H2, 1:2000 dilution), GAPDH (Boster, China; BM0627, 1:1000 dilution), MyoD (Santa Cruz Biotechnology; sc-377,186, 1:200 dilution), Akt (Cell Signaling Technology, Danvers, MA, USA; 40D4, 1:2000 dilution), pAkt (Cell Signaling Technology, Danvers, MA, USA; D9E, 1:1000 dilution), carboxyl-terminal modulator protein (CTMP) (Santa Cruz Biotechnology, CA, sc-390,353 1:200 dilution), PTEN (Cell Signaling Technology, Danvers, MA, USA; Y184, 1:1000 dilution), and phospho-CREB at ser133 rabbit monoclonal (Cell Signaling Technology, Danvers, MA, USA; 87G3), followed by incubation with goat anti-mouse IgG-horseradish peroxidase (HRP) (Santa Cruz Biotechnology; sc-2005, 1:3000 dilution) and goat anti-rabbit IgG-HRP (Santa Cruz Biotechnology; sc-2004, 1:3000 dilution) secondary antibodies for 1 h at room temperature. Immune complexes were detected using the Clarity™ and Clarity Max™ Western ECL Blotting Substrates (BIORAD, California, USA). The band signal intensities were determined using ImageJ software. Western blots were quantified by normalized the band density of phosphorylated proteins to their total protein level, whilst all other proteins were quantified by normalized the band to GAPDH.

### Immunofluorescence

Immunofluorescence was performed as previously described [36]. The primary antibodies included mouse anti-MyHC (1:100 dilution) and NDRG4 (1:50 dilution). The secondary antibodies included anti-rabbit FITC-IgG (Beyotime, Jiangsu, China) and anti-mouse Cy3-IgG (Beyotime). Myoblast fusion was analyzed by calculating numbers of nuclei in MyHC-positive cells. The number of nuclei in individual myotubes was counted for 50-100 myotubes in 8 random fields. MyHC-positive cells were grouped into: those with one nuclei, those with 2-5 nuclei, and those with ≥6 nuclei. The percentages of nuclei in these three groups were expressed as percentages of total nuclei in MyHC-positive cells.

### Co-immunoprecipitation assay

C2C12 myoblasts were seeded in 10-cm dishes and differentiated for 3 days. Cells were lysed in 1 ml lysis buffer (Sangon, Shanghai, China) and protease inhibitor (Sangon). The lysate was centrifuged to remove insoluble components and incubated with either anti-NDRG4 antibody, anti-CTMP antibody, or IgG antibody (Beyotime) overnight at 4°C. The immune complexes were then incubated with Protein A + G agarose beads (Beyotime) for 1.5 h. The beads were washed five times with lysis buffer. The proteins were analyzed by Western blotting as described above.

### Chromatin immunoprecipitation (ChIP) assay

ChIP assays were performed using the ChIP Assay Kit (Beyotime). Briefly, after crosslinking the chromatin with 1% formaldehyde at 37°C for 20 min and neutralizing with glycine for 5 min at room temperature, C2C12 myoblasts were washed with cold phosphate-buffered saline, scraped, and collected. Nuclear lysates were sonicated 15 times for 10 s at 10-s intervals on ice using a Sonics VCX 130 (Sonics, USA). The chromatin complex was immunoprecipitated at 4°C overnight with pCREB (Ser133) rabbit antibodies using phenol/chloroform. Real-time PCR was performed to analyze the DNA fragments. The primers were MyoD-cF (forward primer, 5’-CCCAGATGGAGAATGACCAAA-3’), MyoD-cR (reverse primer, 5’- AAGGCTACGGGACAATGAAAG-3’) and MyoG-cF (forward primer, 5’-TCAAAGAAGCTGTAGAAACCCAA-3’), MyoG-cR (reverse primer, 5’-TCAGCAG CACCTTAAACCATAC-3’). According to the ChIP analysis manual (Thermofisher scientific), Relative enrichment is calculated as the amount of amplified DNA normalized to input and relative to values obtained after normal IgG immunoprecipitation.

### Statistical analysis

All results are presented as the means ± standard error. The data were accumulated from at least three independent experiments. Two-tailed Student's *t*-tests were used for *P*-value calculations.
